# Invasive Urogenital *Neisseria meningitidis* Serogroup Y Multilocus Sequence Type 1466

**DOI:** 10.3201/eid3110.250902

**Published:** 2025-10

**Authors:** C.R. Robert George, Hemalatha Varadhan, Syeda Naqvi, Brett Locker, Matthew Howes, Sebastiaan van Hal, Monica M. Lahra

**Affiliations:** New South Wales Health Pathology, New Lambton Heights, New South Wales, Australia (C.R.R. George, H. Varadhan, S. Naqvi); John Hunter Hospital, New Lambton Heights (B. Locker, M. Howes); New South Wales Health Pathology, Camperdown, New South Wales, Australia (S. van Hal); The University of Sydney, Camperdown (S. van Hal); New South Wales Health Pathology, Randwick, New South Wales, Australia (M.M. Lahra); The University of New South Wales, Randwick (M.M. Lahra).

**Keywords:** *Neisseria meningitidis*, urogenital infections, bacteria, bacterial infections

**To the Editor:** Coincident outbreaks of *Neisseria meningitidis* serogroup Y (MenY) multilocus sequence type (ST) 1466 were detected in 2024, manifesting as invasive meningococcal disease (IMD) in the United States and urogenital infections in Australia (*1*). We read with interest the genomic analysis of globally available MenY ST1466 sequences that found that isolates from outbreaks in the United States and Australia were closely related (*2*). Although the study provided evidence that the urogenital site could act as a reservoir for IMD cases, risk estimates of urogenital MenY ST1466 ability to cause IMD or the propensity for the invasive isolates to inhabit the urogenital niche were lacking (*2*). Here, we report the case of a previously healthy 29-year-old woman who sought care at an emergency department for postprocedural pelvic inflammatory syndrome 3 days after the insertion of an intrauterine device. 

We collected microbiological samples as the initial case workup. Vaginal swab specimens were negative for *Chlamydia trachomatis* and *N. gonorrhoeae*. Oropharyngeal specimens were not collected. MenY was isolated from a positive blood culture, and subsequent investigation included additional vaginal samples for culture. Although culture results were negative, MenY was detected by PCR from 5 vaginal and intrauterine device fluid samples. We conducted whole-genome sequencing on the blood culture after DNA extraction by using Illumina iSeq (Illumina, https://www.illumina.com), according to manufacturer instructions. In silico typing confirmed MenY ST1466. The patient was treated with ceftriaxone and made a full recovery.

This case represents presumptive progression of urogenital carriage of MenY ST1466 to invasive disease, supporting the premise that urogenital carriage is a risk factor for IMD (*2*). On the basis of the link between upper airway inflammation associated with influenza and IMD, it is biologically plausible that meningococcal urethritis similarly poses an increased risk for IMD (*3*). Concerns remain in Australia regarding the risk posed by urogenital MenY cases being detected through enhanced laboratory practices.
